# Light- and Temperature-Induced Expression of an R2R3-MYB Gene Regulates Anthocyanin Biosynthesis in Red-Fleshed Kiwifruit

**DOI:** 10.3390/ijms20205228

**Published:** 2019-10-22

**Authors:** Min Yu, Yuping Man, Yanchang Wang

**Affiliations:** 1 Key Laboratory of Plant Germplasm Enhancement and Specialty Agriculture, Wuhan Botanical Garden, Chinese Academy of Sciences, Wuhan 430074, China; yumindsd@163.com (M.Y.); manyuping@126.com (Y.M.); 2 College of Life Sciences, University of Chinese Academy of Sciences, Beijing 100049, China

**Keywords:** *AcMYB10*, kiwifruit, R2R3-MYB, anthocyanin, light, temperature

## Abstract

The R2R3 MYB genes associated with the flavonoid/anthocyanidin pathway feature two repeats, and represent the most abundant classes of MYB genes in plants; however, the physiological role and regulatory function of most R2R3 MYBs remain poorly understood in kiwifruit (*Actinidia*). Here, genome-wide analysis identified 155 R2R3-MYBs in the ‘Red 5′ version of the *Actinidia chinensis* genome. Out of 36 anthocyanin-related AccR2R3-MYBs, *AcMYB10* was the most highly expressed in inner pericarp of red-fleshed kiwifruit. The expression of *AcMYB10* was highly correlated with anthocyanin accumulation in natural pigmentation during fruit ripening and light-/temperature-induced pigmentation in the callus. *AcMYB10* is localized in the nuclei and has transcriptional activation activity. Overexpression of *AcMYB10* elevates anthocyanin accumulation in transgenic *A. chinensis*. In comparison, *A. chinensis* fruit infiltrated with virus-induced gene silencing showed delayed red coloration, lower anthocyanin content, and lower expression of *AcMYB10*. The transient expression experiment in *Nicotiana tabacum* leaves and *Actinidia arguta* fruit indicated the interaction of *AcMYB10* with *AcbHLH42* might strongly activate anthocyanin biosynthesis by activating the transcription of *AcLDOX* and *AcF3GT*. In conclusion, this study provides novel molecular information about R2R3-MYBs in kiwifruit, advances our understanding of light- and temperature-induced anthocyanin accumulation, and demonstrates the important function of *AcMYB10* in the biosynthesis of anthocyanin in kiwifruit.

## 1. Introduction

MYB transcription factors (TFs) are found in a wide range of higher plants and constitute one of the largest transcription factor families. They are characterized by a structurally conserved DNA-binding domain that consists single or multiple imperfect repeats located near the N-terminus. These repeats function synergistically or individually in DNA binding and protein–protein interactions, respectively [1]. In plants, MYB-containing genes have greatly diversified, being classified into four major subfamilies (1R-, R2R3-, 3R-, and 4R-MYB) based on the number of adjacent MYB repeats [2]. R2R3-MYB proteins probably evolved from an R1-MYB gene through the duplication of an R1 repeat or from an R1R2R3-MYB gene through the loss of an R1 repeat, and form the largest subfamily of MYB transcription factors in the MYB super family [2]. For example, 198 MYB genes were identified in *Arabidopsis thaliana*, of which 126 belong to R2R3-MYB [3]. Furthermore, 88 and 244 R2R3-MYB proteins have been identified in rice (*Oryza sativa*) and soybean (*Glycine max*), respectively [4,5]. R2R3-MYB proteins play an important role in regulatory networks that are involved in metabolic, cellular, and developmental processes, as well as responses to biotic and abiotic stresses [3]. The first plant MYB gene, *C1*, was isolated from maize (*Zea mays*), and encodes a c-MYB-like protein involved in the anthocyanin biosynthesis pathway [6]. More recently, R2R3-MYBs were reported to contribute to regulation of anthocyanin accumulation in *Arabidopsis*, grape (*Vitis vinifera*), Asian pear (*Pyrus pyrifolia*), and apple (*Malus domestica*) [7,8,9,10].

Kiwifruit, namely the genus *Actinidia*, is represented by 54 species and 75 taxa [11] and is one of the most valuable fruit crops worldwide (5,287,605 tons in 2014, http://www.fao.org/). Among the kiwifruit taxa, red pigmentation is the result of the accumulation of anthocyanins and only occurs in a few species, such as *Actinidia chinensis*, *Actinidia arguta*, *Actinidia deliciosa,* and *Actinidia eriantha* [12]. For this reason, red-fleshed kiwifruit cultivars, such as *A. chinensis* cv. Hongyang, have attracted increasing interest by consumers because of its excellent fruit quality and health properties attributed to anthocyanins [13,14]. The genes associated with anthocyanin metabolism in kiwifruit have been studied over the last decade. *AcF3GT1* is a key structural gene in the anthocyanin biosynthesis pathway and is required for anthocyanin biosynthesis in red-fleshed *A. chinensis* [15]. R2R3-MYBs are also involved in the biosynthesis of anthocyanin in kiwifruit. *AcMYB110a* determines the red color of the petal and activates *F3GT1* to induce anthocyanin accumulation in *A. erientha* [16]. In ‘Hongyang’, the expression of *AcMYB75* is closely related to anthocyanin accumulation during fruit development [17]. Transient color assays reveal that *AcMYBF110* can autonomously induce anthocyanin accumulation in *N. tabacum* leaves by activating the transcription of *NtDFR, NtANS,* and *NtUFGT* [18]. Using both transient assays in *N. tabacum* leaves or *A. arguta* fruits and stable transformation in *Arabidopsis*, a previous report demonstrated that co-expression of *AcMYB123* and *AcbHLH42* is a prerequisite for anthocyanin production by activating transcription of *AcF3GT1* and *AcANS* [19].

Anthocyanin-related R2R3-MYB is closely regulated by environmental factors such as light and temperature [20]. In *A. thaliana*, anthocyanin biosynthesis is influenced by different light qualities through MYB (*PAP1* and *PAP2*) and bHLH (*TT8*, *EGL3*, and *GL3*) [21]. Accumulation of anthocyanins in response to light is mediated by R2R3 MYB transcription factors in many fruit crops, including apple, pear, litchi, eggplant, and bayberry [22,23,24,25,26]. In addition to light, temperature is an important factor that affects anthocyanin biosynthesis in plants. High temperatures decrease anthocyanin content in the skin of apple and grapevine berries [27,28]. Light and temperature also have a synergistic effect on anthocyanin accumulation in fruit. For instance, in grape berry, low temperature and light intensity have a synergistic effect on the expression of genes in the flavonoid biosynthesis pathway in the skin [27]. In red-fleshed apple, light and temperature are important factors involved in the anthocyanin biosynthesis of callus cultures, by regulating the expression of MYBs [29]. In kiwifruit, the up-regulation of *MYBA1-1* and *MYB5-1* by low temperature could effectively enhance anthocyanin accumulation in kiwifruit during storage, probably through the transcriptional activation of *ANS1*, *ANS2*, *DFR1*, *DFR2*, and *UFGT2* [30]. By contrast, high temperature suppresses *AcMYB1* expression, which contributes to reduce anthocyanin accumulation in kiwifruit [31]. However, the molecular mechanisms underlying the combined effects of light and temperature on anthocyanin biosynthesis have not been comprehensively investigated in kiwifruit.

This study aimed to characterize the candidate R2R3-MYBs involved in the accumulation of anthocyanin in kiwifruit and to understand how the regulatory network of anthocyanin biosynthesis is affected by light and temperature. The results are expected to allow us to elucidate the function of the key MYBs responsible for anthocyanin accumulation in kiwifruit. Our findings are also expected to provide new insights on the functions of MYB genes in red-flesh kiwifruit, providing a baseline for detecting genes of significance for genetic manipulation.

## 2. Results and Discussion

### 2.1. Identification, Genomic Location, Gene Structure, and Motif Composition of R2R3-MYB Genes in Kiwifruit

In total, 224 deduced amino acid sequences that contain MYB or MYB-Like domains were identified in the “Red5” kiwifruit genome. Among these, 155 R2R3-MYB (AccR2R3-MYB) were identified based on a conserved domain search, and proteins shared 108 conserved R2R3 residues. From the alignment, a series of periodic Trp (W) residues was found in each MYB repeat, which were considered as a landmark of the MYB domain. In addition to the highly conserved tryptophan residues, Asp-14, Cys-45, and Arg-48 in R2 repeat; Leu-53 in the linker region; and Arg-91 and Thr-92 in the R3 repeat were also completely conserved in all of the AccR2R3-MYB proteins (Supplementary Figure S1A).

The 155 AccR2R3-MYB genes were unevenly distributed across all 29 chromosomes, except chromosome 19 (Supplementary Figure S1B). Chromosome 23 harbored the most abundant (13) AccR2R3-MYBs, followed by chromosomes 14 and 18 (10). In comparison, only two AccR2R3-MYBs were found on chromosomes 2, 4, 6, 10, and 21, respectively (Supplementary Figure S1B).

To investigate the conserved motifs in this family, the 155 AccR2R3-MYBs were subjected to MEME Suite 5.0.5 (Supplementary Figure S2). All of these 155 AccR2R3-MYB contained 1#, 2#, and 3# motifs, which, together, constructed the conserved R2R3 domain. Most of the phylogenetically close members had common conserved motifs, indicating that they had similar biological functions.

The genomic length of AccR2R3-MYB ranged from 970 bp (Acc27824) to 10,640 bp (Acc15993) (Supplementary Figure S2). 126 AccR2R3-MYB genes contained three exons, 25 contained two exons, three (Acc02688, Acc29660, and Acc14973) contained four exons, and only Acc27824 contained one exon. Most of these AccR2R3-MYBs contained one to two introns.

### 2.2. Phylogenetic Analysis of AccR2R3-MYB TFs

To subclassify the AccR2R3-MYBs, a neighbor-joining (NJ) phylogenetic tree was constructed using the R2R3 conserved domain of the 155 AccR2R3-MYBs and 126 AtR2R3-MYBs (Supplementary Figure S3). According to the tree topology, bootstrap values, and the previous classification of AtR2R3-MYBs [3,32], we classified the 155 AccR2R3-MYBs into 27 subgroups (designated C1–C27 in Supplementary Figure S3). The largest group (C16) contained 14 members, followed by C11, which contained 13 members. In comparison, group C27 was the smallest, containing only one AccR2R3-MYB member. Among the 27 groups, C9 to C14 were in a same clade, containing *AtMYB75, AtMYB90, AtMYB113,* and *AtMYB114* (highlighted in red in Supplementary Figure S3), which are involved in anthocyanin biosynthesis in *Arabidopsis* [7]. Acc10232, Acc00493, and Acc10227 had a very close phylogenetic relationship with these anthocyanin-related *AtMYBs*, indicating that their functions were related. More importantly, Acc10232 (*AcMYB110*) determined anthocyanin accumulation in the kiwifruit petal [16]. Thus, the 36 AccR2R3-MYBs in C9 to C14, especially Acc10232 (*AcMYB110*), Acc00493 (*AcMYB10*), and Acc10227, are probably involved in anthocyanin biosynthesis in kiwifruit.

### 2.3. Expression Preference of AccR2R3-MYBs in Kiwifruit

**Figure 1:**
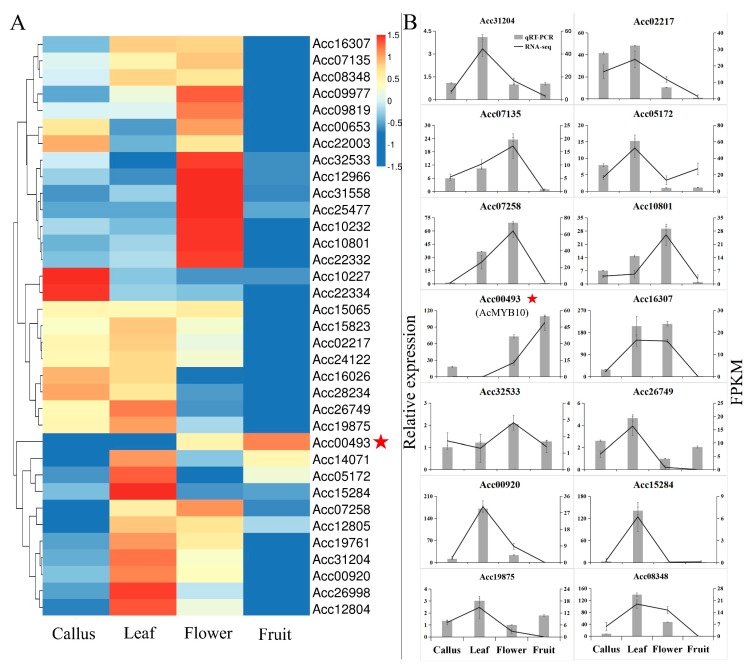
Tissue expression profiles of 36 anthocyanin-related AccR2R3-MYB genes in kiwifruit. (**A**) Hierarchical clustering of expression profiles of 36 candidate AccR2R3-MYBs in different tissues. Log2(FPKM+1) (Fragments per kilobase of transcript per million mapped reads) values were displayed according to the color code (top right). (**B**) Expression analysis of selected genes using quantitative real-time RT-PCR in different tissues. *AcMYB10* (Acc00493) was marked with a red pentagram.

The expression preference of the identified AccR2R3-MYBs was investigated in four tissues, including the callus, leaf, flower, and inner pericarp using RNA-seq data (Supplementary Table S1). The expression levels of 155 R2R3-MYBs were clustered into four main groups, as shown in Supplementary Figure S3. Of note, all of the 155 AccR2R3-MYBs showed various tissue preferences, and their expression levels were not clustered in the phylogenetic tree (Supplementary Figure S4). Thus, the expression levels of the 36 anthocyanidin-related MYBs were exclusively clustered again (Figure 1A). In addition, only one gene, Acc00493 (*AcMYB10*), was clustered alone, and showed an expression preference for the inner pericarp of fruit, indicating that it is involved in the fruit pigmentation of kiwifruit. To verify the RNA-seq data, quantitative real-time RT-PCR was performed on four different tissues for 14 selected AccR2R3-MYBs. As shown in Figure 1B, the genes showed very distinct tissue-specific expression patterns, which were in good agreement with the RNA-seq data.

### 2.4. Identification of Cis-Acting Regulatory Elements of Anthocyanin-Related AccR2R3-MYBs

**Figure 2:**
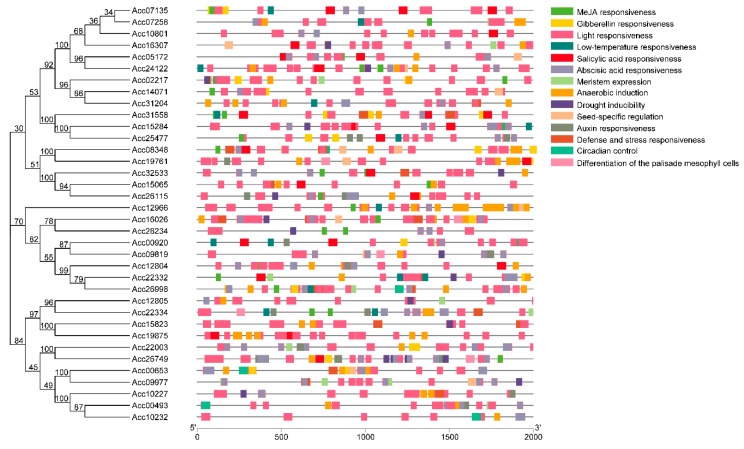
Distribution of cis-acting regulatory elements (CREs) involved in the responsiveness and development of the promoters of 36 candidate AccR2R3-MYBs.

To establish the “regulation–expression” cue of these candidate AccR2R3-MYBs, the cis-acting regulatory elements (CREs) located at 2000 bp upstream of the 36 genes (designated C1–C27 in Supplementary Figure S3) were identified using the PlantCARE database (Supplementary Table S2). A total of 3405 CREs were identified and classified into six groups based on their annotations, which included ‘cellular regulation’ (72.3%), ‘light responsiveness’ (14.2%), ‘hormone signaling’ (7.4%), ‘biotic and abiotic stresses’ (4.0%), ‘developmental processes’ (1.5%), and ‘biosynthetic processes’ (0.7%). In the ‘light responsiveness’ group, most of these CREs were allocated to ‘G-Box’ (127) and ‘Box 4’ (97). The ‘biotic and abiotic stresses’ group contained 135 CREs, of which 15 were allocated to low-temperature stress (LTR). The distribution of CREs involved in responsiveness and development is shown in Figure 2. ‘Light responsiveness’ was the dominant CRE that was distributed in all of the promoters. However, the distribution of the CRE in these promoters was highly diverse, but not conserved. Specifically, there was no parallel conservation that was correlated to the phylogenetic tree constructed based on the coding sequences (CDS) of the 36 anthocyanidin-related MYBs (Supplementary Figure S3). Thus, these MYBs have various regulatory patterns, indicating that they evolved quickly with high functional differentiation. This result supports the pattern of expression for these MYBs shown in Figure 1A. Thus, combined with previous studies on the regulation of MYBs correlated to anthocyanidin accumulation by environmental factors [20], we speculated that the expression of anthocyanin-related AccR2R3-MYBs would also respond to light or temperature in specific tissues.

### 2.5. Light and Low-Temperature Synergistically Induced the Red Pigmentation in the Callus of A. chinensis cv. Hongyang

**Figure 3:**
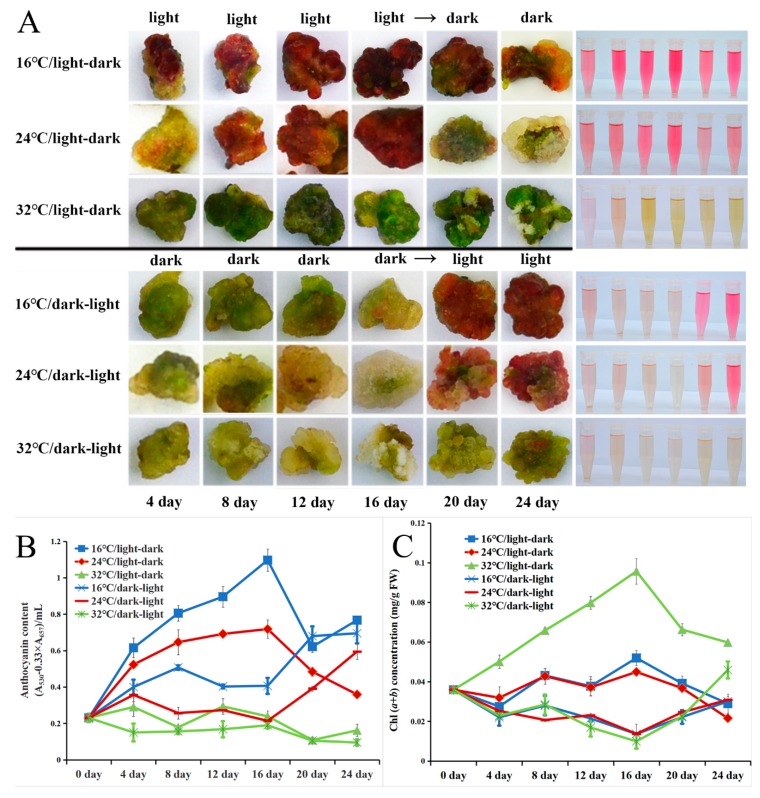
Relative callus anthocyanin content and chlorophyll content under different temperatures and light conditions. (**A**) Changes to callus color under different conditions and culture stages. The tubes in the right part contain the fresh extracts from left callus. (**B**) Anthocyanin content under different culture conditions over 24 days. (**C**) Chlorophyll (Chl *a* + Chl *b*) content over 24 days. Results represent the mean ± SD of three replicates.

To investigate the effect of light and temperature on anthocyanin accumulation in kiwifruit, the callus of ‘Hongyang’ was cultured at different temperatures and light/dark conditions. As shown in Figure 3A, under the light-to-dark condition, the red pigment gradually accumulated in the 16 °C and 24 °C treatments when cultured under days with continuous light conditions. Then, the redness of the callus gradually faded after transition to dark at 16 °C and immediately faded at 24 °C. Only a little pigment appeared on the callus at 32 °C treatment under light or dark conditions. Under the dark-to-light condition, all the callus remained green/pale-green under continuous darkness. However, the callus immediately became red after exposure to light at 16 °C and 24 °C. In comparison, at 32 °C, the callus showed no obvious color change during the whole process. Anthocyanin content was determined in all of these samples (Figure 3B). Variation in anthocyanin levels in every treatment of the callus was strongly consistent with the level of red pigmentation, thus leading to the anthocyanin biosynthesis of kiwifruit responses to light and low temperature. This suggestion supports previous reports about the synergistic effects of light and temperature on anthocyanin accumulation in grape skin and apple callus [27,29]. High temperature might inhibit (light-induced) anthocyanin biosynthesis or promote anthocyanin degradation in kiwifruit, as reported for grape [33]. 

Of note, the green pigmentation changed regularly with treatment in our study (Figure 3A). Previous studies reported that the accumulation of both chlorophyll and anthocyanin is affected by light and temperature [27,34], with the green pigment (chlorophyll) showing opposite accumulation trends to the red pigment (anthocyanin) during fruit ripening [35]. Thus, we investigated the chlorophyll content of all callus samples. As shown in Figure 3C, under the light-to-dark condition, chlorophyll content increased under continuous light and sharply decreased after light/dark transition, showing the same trend as anthocyanin content (except at 32 °C). In comparison, under the dark-to-light condition, chlorophyll content remained low and gradually decreased, but increased immediately after exposure to light, which showed the same trend as anthocyanin content (except at 32 °C). Thus, both chlorophyll and anthocyanin in the kiwifruit callus accumulate in response to light. In particular, relatively high temperature (32 °C) accelerates the accumulation of chlorophyll but inhibits the accumulation of anthocyanin, and vice versa. In brief, temperature might have contrasting effects on anthocyanin and chlorophyll accumulation. However, the interaction between anthocyanin and chlorophyll biosynthesis during fruit ripening requires further investigation.

**Figure 4:**
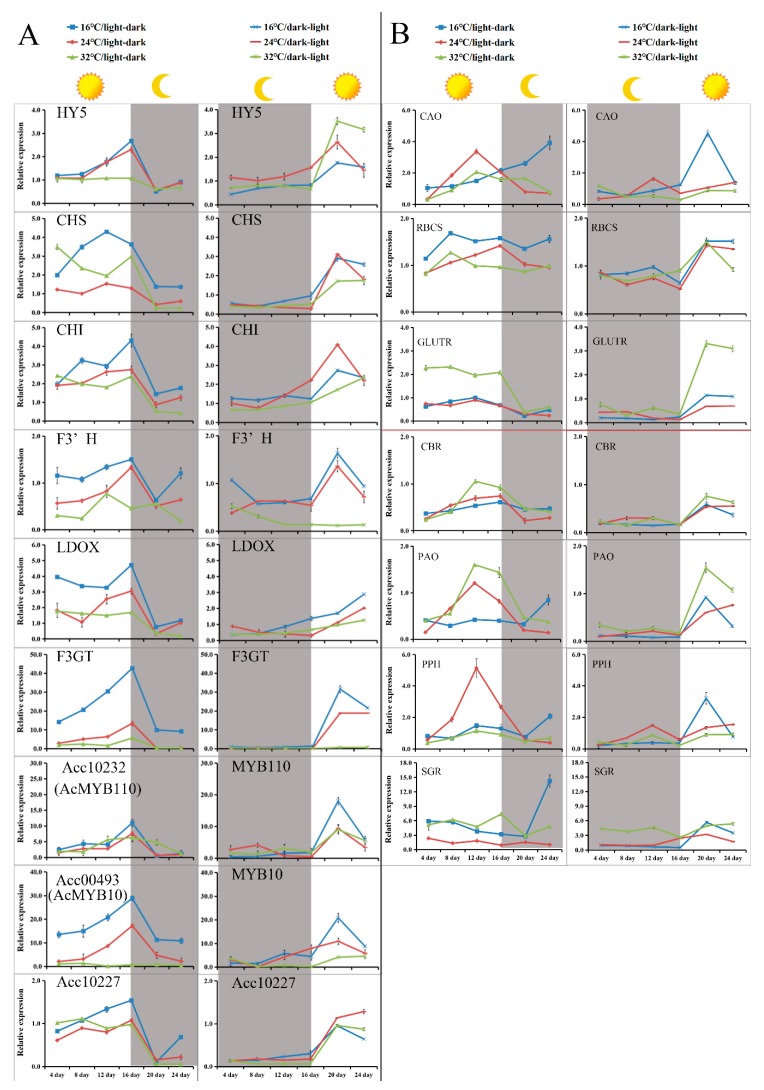
Relative expression levels of anthocyanin- and chlorophyll-related genes in the callus over 24 days under different culture conditions. (**A**) Relative expression levels of the structural genes and AccR2R3-MYBs in the anthocyanin biosynthesis pathway. *HY5*, LONG HYPOCOTYL5; *CHS*, Chalcone synthase; *CHI*, chalcone isomerase, *F3′H*, flavanone 3′-hydroxylase; *LDOX*, leucoanthocyanidin dioxygenase; *F3GT*, UDP flavanone- 3-Oglucosyltransferase. (**B**) Expression levels of genes related to chlorophyll metabolism. *CAO*, Chlorophyll *a* oxygenase; *RBCS*, Small subunit of ribulose-1,5-bisphosphate Carboxylase; *GLUTR*, Glutamyl tRNA reductase; *CBR*, Chlorophyll b reductase; *PAO*, Pheophorbide a oxygenase; *PPH*, Pheophytin pheophorbide hydrolase; *SGR*, Stay-green. Results represent the mean ± SD of three replicates.

To evaluate the genetic basis of these phenotypes, the expression levels of five structural genes on the anthocyanin biosynthesis pathway (*CHS*, *CHI*, *F3′H*, *LDOX,* and *F3GT*), three AccR2R3-MYBs (*AcMYB110*, *AcMYB10*, and Acc10227), and *HY5* (LONG HYPOCOTYL5, a positive transcription factor that responds to light and is involved in anthocyanin accumulation) [36] were investigated using qRT-PCR. As shown in Figure 4A, after light/dark transition, the expression level of all of these genes noticeably decreased and was then maintained at a relatively low level. After the dark/light transition, the expression levels of all of these genes noticeably increased (and peaked) and then declined slightly but remained at a relatively high level. Thus, the expression of these genes might respond to light. Of note, the expression of *LDOX*, *F3GT,* and *MYB10* was higher at 16 °C compared to 24 °C and 32 °C, indicating temperature sensitivity. *AcF3GT* encodes an enzyme dedicated for anthocyanin biosynthesis in the kiwifruit inner pericarp [15]. Among the expression data, that of *AcMYB10* was similar to that of *AcF3GT*, with their expression curves fitting well, Therefore, *AcMYB10* might contribute to the accumulation of anthocyanin in the kiwifruit callus by regulating *AcF3GT*.

The expression levels of genes related to chlorophyll metabolism (*CAO*, *RBCS, GLUTR*, *CBR*, *PAO*, *PPH,* and *SGR*) were also investigated (Figure 4B). The expressions of *GLUTR*, *CBR,* and *PAO* were higher under light conditions compared to dark conditions. The expression of *GLUTR* was much higher at 32 °C compared to 24 °C and 16 °C. Thus, *GLUTR* expression responds to temperature, promoting chlorophyll biosynthesis in the kiwifruit callus. However, the other six genes showed no similar expression patterns corresponding to chlorophyll content. Thus, a more complicated mechanism, other than light and temperature, likely regulates the green pigmentation of kiwifruit.

### 2.6. Sequence Analysis, Transcriptional Activation Ability, and Expression Characteristic of Acc00493 (AcMYB10)

**Figure 5:**
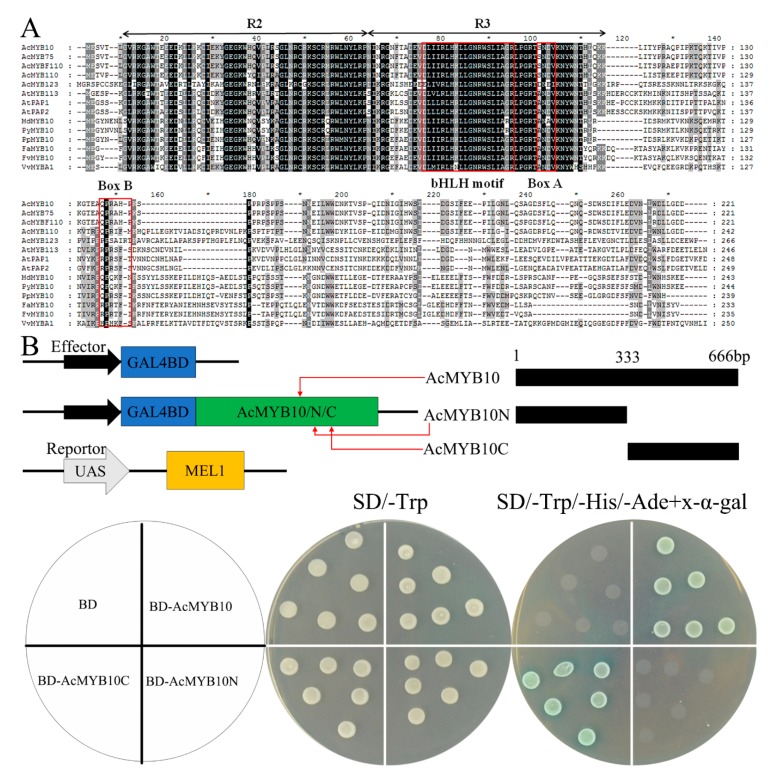
Analyses of the *AcMYB10* activation domain. (**A**) Amino acid sequence alignment of Acc00493 (*AcMYB10*) and other plant anthocyanin-promoting MYBs. Totally conserved residue is highlighted in black and partial conserved is highlighted in gray. R2 and R3 MYB motifs are indicated by arrows. The bHLH motif indicates the residues needed for the interaction with the bHLH partner; box A and box B are well-conserved in anthocyanin-promoting MYBs. The asterisks indicate the number of amino acid. The GenBank accession numbers are: AcMYB75 (APZ74276.1), AcMYBF110 (AYJ72555.1), AcMYB110 (AHY00342.1), AcMYB123 (QAT77713.1), AtMYB113 (NP_176811.1), AtPAP1 (Q9FE25.1), AtPAP2 (Q9ZTC3.1), MdMYB10 (ABB84753.1), PyMYB10 (ADN26574.1), PpMYB10 (ADK73605.1), FaMYB10 (ABX79947.1), FvMYB10 (ABX79948.1), VvMYBA1 (AGH68552.1). (**B**) Transcriptional activity assay of *AcMYB10*. Schematic of the full length and three truncated fragments (AcMYB10N and AcMYB10C) of *AcMYB10* used for constructing vectors. Effectors and reporter used for transcriptional activation activity assay. Growth of yeast cells (strain *AH109*) transformed with each of the three vectors or pGBKT7 empty vector (used as a negative control) on SD/-Trp or SD/-Trp/-His/-Ade, with the addition of X-α-gal. The layout of the effectors is shown in the pie chart on the left.

From above, Acc00493 showed a close phylogenetic relationship with the R2R3-MYBs responsible for anthocyanin biosynthesis in *Arabidopsis*. In addition, it was strongly expressed in the inner pericarp of red-fleshed kiwifruit, and its expression level was correlated with light-/temperature-regulated pigmentation in the kiwifruit callus. Thus, Acc00493 was screened as a candidate gene involved in the biosynthesis of anthocyanin in kiwifruit. To investigate the de novo function of Acc00493, we cloned the full-length CDS of Acc00493 from ‘Hongyang’ genomic DNA. The coding sequence of Acc00493 is identical to *AcMYB10* (Genbank: MG581953.1), but has one nucleotide difference with *AcMYB75* (Genbank: KX349735.1) (Figure 5A), suggesting both *AcMYB10* and *AcMYB75* should probably be designated to locus Acc00493. Since this SNP (single nucleotide polymorphism) will cause a change in one amino acid of the protein sequence, we further amplificated the full-length DNA of Acc00493 in ‘Hongyang’. No *AcMYB75* was detected using either PCR products or TA clones (Supplementary Figure S5), indicating only one allele, *AcMYB10*, is at this locus in ‘Hongyang’ (Supplementary Figure S1B). Because *AcMYB75* was identified using an RNA-seq dataset, and no DNA sequence and flanking sequence of *AcMYB75* have been reported, we speculate that *AcMYB75* is probably a different allele of Acc00493 in another cultivar, but not ‘Hongyang’, or a paralog of Acc00493, which had not been assembled in the present two *A. chinensis* genomes, or just a sequencing or assembly error. Thus, we designated Acc00493 as *AcMYB10* from here on. 

The *AcMYB10* protein contained a highly conserved N-terminal R2R3 repeat of a DNA-binding domain with a bHLH motif which was functionally important for the interaction between MYB and R/B-like bHLH proteins [37] (Figure 5A). To determine the transcriptional activation activity of *AcMYB10*, the full-length and two truncated CDS of *AcMYB10* (*AcMYB10N*[1–111aa] and *AcMYB10C*[112–222aa]) were fused to pGBKT7 to generate three effectors. The effectors were separately transferred to the yeast containing a MEL1 reporter (Figure 5B). All of the yeast cells grew well on the SD medium lacking tryptophan (SD/–Trp), whereas only yeast cells transformed with the effectors containing BD-AcMYB10 or BD-AcMYB10C grew and displayed GAL4 activity on the medium supplemented with X-α-gal (Figure 5B). These results demonstrate that the transcriptional activation activity of *AcMYB10* and the AcMYB10C region is necessary for transactivation. Thus, the C-terminal region within *AcMYB10* may contain key residues that were important for transactivation efficiency, as previously reported [38].

To investigate the expression pattern of *AcMYB10* during the fruit development of red-fleshed kiwifruit, four stages of the red-fleshed *A. chinensis* cv. Hongyang and yellow-fleshed *A. chinensis* cv. Jinyan fruits were sampled for expression and anthocyanin analysis. As shown in Supplementary Figure S6, low anthocyanin levels and low expressions of *AcLDOX*, *AcF3GT,* and *AcMYB10* were detected in the inner pericarp of ‘Jinyan’ during fruit development. In comparison, an increasing anthocyanin content and expression of *AcMYB10*, *AcF3GT,* and *AcLDOX* were detected in the inner pericarp of ‘Hongyang’ along with fruit development and pigmentation. These results support that anthocyanin accumulation in the inner pericarp is associated with the coordinated action of *AcMYB10*, *AcF3GT,* and *AcLDOX* during fruit development and ripening, supporting previous work [17].

### 2.7. Gene Functional Study by Overexpression of AcMYB10 in A. chinensis cv. Hongyang and Using the VIGS (Virus-Induced Gene Silencing) System in A. chinensis cv. Hongshi No. 2

**Figure 6:**
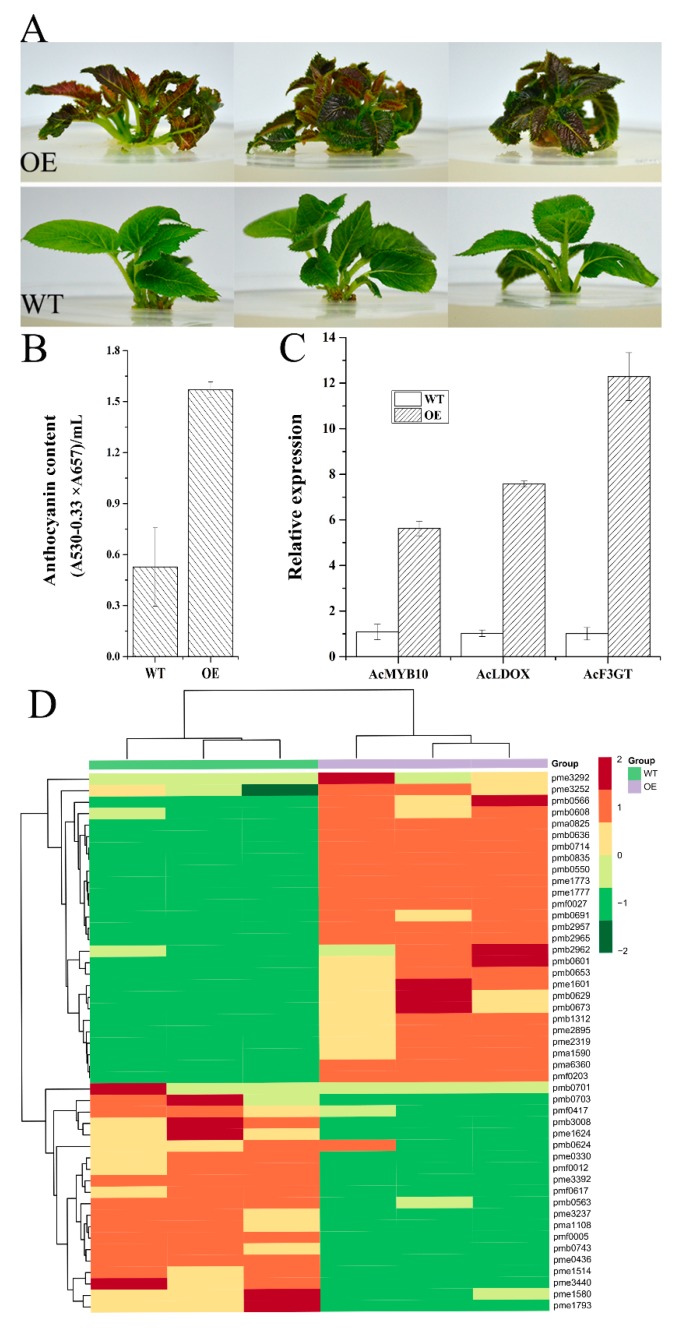
Wild type (WT) and transgenic ‘Hongyang’ (OE) expressing *AcMYB10* under the control of the 35S promoter. (**A**) Phenotypes of transgenic and wild-type ‘Hongyang’ kiwifruit. (**B**) Anthocyanin content in transgenic kiwifruit leaves. (**C**) Expression level of *AcMYB10*, *AcLDOX*, *AcF3GT* in wild-type and transgenic kiwifruit leaves. (**D**) Heat map visualization of flavonoid metabolites. Each metabolite is represented by a single row. Dark red indicates relatively high abundance, whereas metabolites with relatively low abundance are shown in dark green (color key scale on the right of the heat map).

To further reveal the function of *AcMYB10*, the recombinant vector 35S:AcMYB10 was transformed to the ‘Hongyang’ callus for generating transgenic kiwifruit plants. The leaves of 35S:AcMYB10 (OE) had a clear red color at the young stage, whereas the wild type (WT) showed no obvious abnormal phenotype (Figure 6A). The anthocyanin content of OE lines was significantly higher than that of WT, and the expressions of *AcMYB10*, *AcF3GT,* and *AcLDOX* were also significantly higher in OE compared to WT (Figure 6B,C).

Anthocyanins belong to the flavonoid class of compounds. The accumulation of anthocyanins was associated with multiple upstream flavonoid metabolites [20]. Thus, to investigate metabolite dynamics caused by the over-expression of *AcMYB10* comprehensively, a widely targeted metabolomics method was employed to identify and quantify relative flavonoid metabolites in the leaf of transgenic ‘Hongyang’ (OE) [39]. A total of 224 flavonoids were identified in OE and WT (for details, see Supplementary Table S3). A total of 47 differential metabolites had significantly different contents between OE and WT, including 13 anthocyanins, 2 polyphenols, 1 flavonoid, 5 flavanones, 22 flavones, 1 flavonol, 1 proanthocyanidin, and 2 isoflavones (Figure 6D). Twenty-seven and 20 of these metabolites were up-regulated and down-regulated in OE, respectively (Supplementary Table S3). Of note, all seven identified cyanidin-, two peonidin-, and one pelargonidin-based anthocyanins were up-regulated in OE, whereas two peonidin- and two pelargonidin-based anthocyanins were down-regulated. Because cyanidins are the main anthocyanin in *A. chinensis* responsible for the red color [12], these results indicate that the overexpression of *AcMYB10* promotes the biosynthesis of metabolites in the cyanidin pathway, leading to the red color of transgenic young leaves of OE.

**Figure 7:**
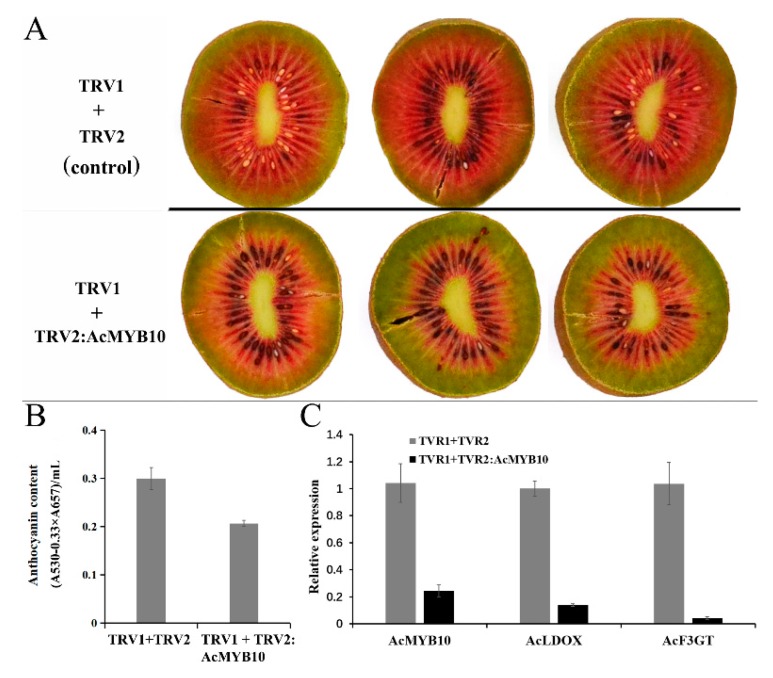
Functional analysis of *AcMYB10* using virus-induced gene silencing (VIGS). (**A**) Silencing of *AcMYB10* in ‘Hongshi No. 2′ fruits. The photograph was taken seven days after infiltration. (**B**) Anthocyanin content of treated fruits. (**C**) Expression level of *AcMYB10*, *AcLDOX* and *AcF3GT* in the inner pericarp of treated fruits.

This study used VIGS on ‘Hongshi No. 2′ fruit to suppress *AcMYB10*. After 7 days, the expression of *AcMYB10* was strongly suppressed in fruit infiltrated with TRV1and TRV2:AcMYB10, leading to delayed pigmentation in infiltrated fruit. In comparison, pigmentation was normal in the fruits infiltrated with TRV1 and TRV2 (Figure 7). In addition, the anthocyanin content was much lower for the fruit treated with TRV1 and TRV2:AcMYB10 compared to fruit treated with TRV1 and TRV2 (Figure 7). Overall, these results demonstrate that *AcMYB10* is involved in anthocyanin accumulation in red-fleshed kiwifruit.

### 2.8. Subcellular Localization Analysis

Because MYBs have been widely reported to interact with bHLH, leading to the activation of structural genes on anthocyanin biosynthesis [40], we investigated the subcellular distribution of the *AcMYB10* protein and *AcBHLH42*. The fusion vector (*AcMYB10*-YFP and *AcBHLH42*-YFP) and the control vector (YFP) were transiently expressed in tobacco leaves, respectively. Confocal imaging of the epidermis showed that just YFP was detected throughout the entire cell, while *AcMYB10*-YFP and *AcBHLH42*-YFP fusion proteins were exclusively localized in the nucleus (Supplementary Figure S7). Localization in the nucleus was confirmed using the co-transformation of a nucleus marker gene fused to mCherry in the epidermis. This result indicates that both *AcMYB10* and *AcBHLH42* are nuclear proteins. Thus, physical interaction between them is possible.

### 2.9. Protein–Protein Interactions between AcMYB10 and AcbHLH42

**Figure 8:**
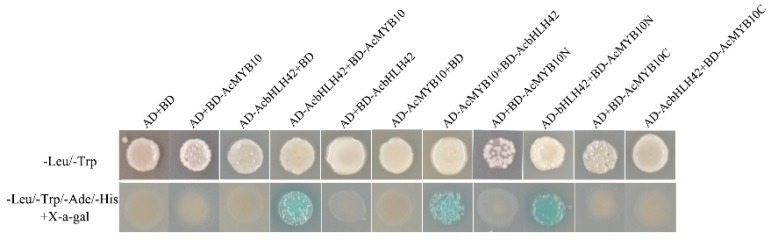
Interactions between *AcMYB10* and *AcbHLH42* detected by the yeast two-hybrid assay. *AH109* yeast cells containing plasmids were grown on double-and quadruple-selection media. The X-α-gal assay was performed to confirm positive interactions.

The yeast two-hybrid assay was employed to demonstrate the interaction between *AcMYB10* and *AcBHLH42* proteins. Yeast harboring *AcBHLH42*-AD + *AcMYB10*-BD and *AcBHLH42*-BD + *AcMYB10*-AD grew well on the quadruple-selection medium. In comparison, the negative control that contained BD + *AcMYB10*-AD, BD + *AcBHLH42*-AD, AD + *AcBHLH42*-BD, and AD + *AcMYB10*-AD did not grow (Figure 8). Thus, a physical interaction occurs between *AcMYB10* and *AcbHLH42* proteins in vitro. Subsequently, two bait vectors encoding different *AcMYB10* regions [AcMYB10N(1–111aa) and AcMYB10C (112–222aa)] fused to the BD and co-transformed to the yeast strain *AH109*, along with the prey vector *AcBHLH42*-AD, to identify the interaction region. The yeast colonies expressing *AcBHLH42* and N-terminal *AcMYB10* truncations (AcMYB10N) grew well on the medium (SD/−Ade/−His/−Leu/−Trp) containing 15 mM 3-amino-1,2,4-triazole (3AT). In comparison, yeast colonies expressing *AcBHLH42* and *AcMYB10* constructs containing the C-terminal region (AcMYB10C) did not grow on the medium (SD/−Ade/−His/−Leu/−Trp) containing 15 mM 3AT. Thus, the N-terminal was essential for protein–protein interactions between *AcMYB10* and *AcBHLH42* (Figure 8). Therefore, the bHLH motif (located on the N-terminal) is likely a key DNA-binding domain for the interaction between *AcBHLH42* and *AcMYB10*, as previously reported [37]. These results suggest *AcMYB10* can interact with *AcBHLH42* and may further promote anthocyanin accumulation in kiwifruit.

### 2.10. AcMYB10 and AcbHLH42 Regulate the Promoter Activities of AcLDOX and AcF3GT

**Figure 9:**
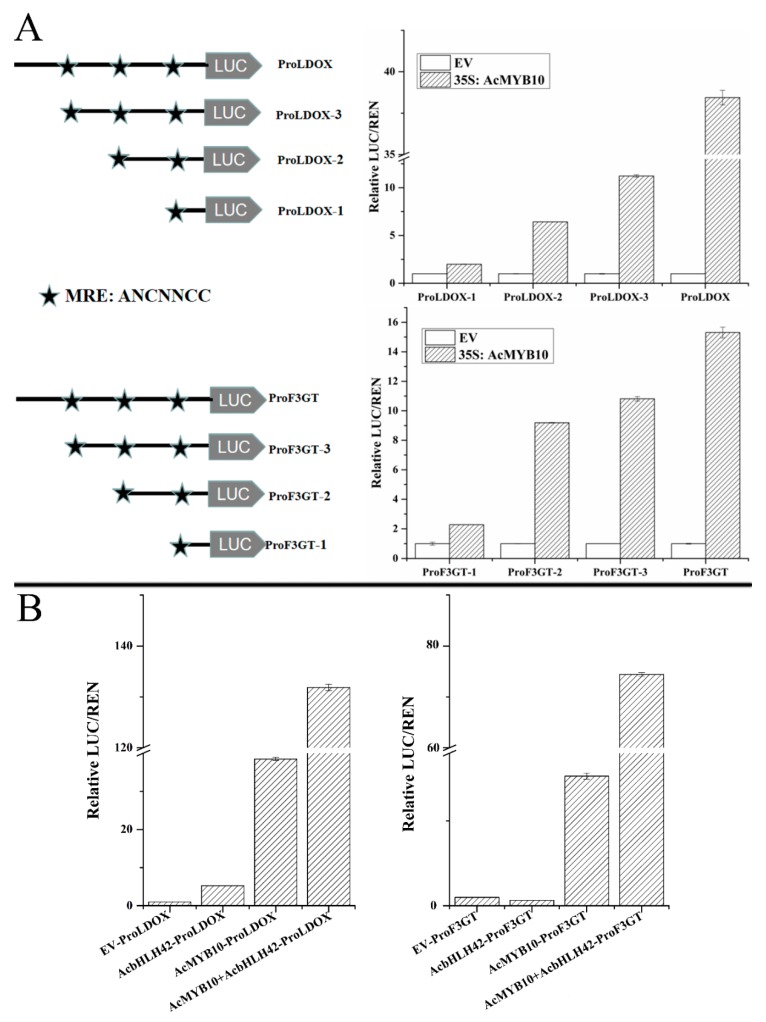
Transcriptional activation analysis of *AcMYB10*-mediated and *AcBHLH42*-mediated induction of *AcLDOX* and *AcF3GT* promoter activities. (**A**) Diagrams showing various DNA fragments of the two selected promoters linked to the firefly luciferase reporter. (**B**) *AcMYB10*-mediated and *AcBHLH42*-mediated induction of *AcLDOX* and *AcF3GT* promoter activities. LUC, Firefly luciferase activity; REN, Renilla luciferase activity. The ratio of LUC/REN of the empty vector (EV) plus promoter was used as a calibrator (set as 1). Results represent the mean ± SD of six replicates.

To evaluate the ability of *AcMYB10* and *AcbHLH42* to activate to the promoter sequences of the anthocyanin biosynthetic genes *AcLDOX* and *AcF3GT* transcriptionally, a dual luciferase system was used on transiently transfected *N. benthamiana* leaves. As shown in Figure 9A, *AcMYB10* activated the promoters of *AcLDOX* (~38 fold compared to the control) and *AcF3GT* (~15 fold). Sequence analysis showed that both *AcLDOX* and *AcF3GT* promoters contain three 7-bp MYB recognizing elements (MREs) (Supplementary Figure S8), which might be the target of MYBs [41]. As shown in Figure 9A, a set of deletion mutation constructs was generated to test the functional roles of these MREs. As expected, each MRE deletion on *AcLDOX* and *AcF3GT* promoters partially reduced the transactivation of *AcLDOX* and *AcF3GT* caused by *AcMYB10*. The deletion of fragments that did not contain MRE also reduced transactivation; thus, other recognition elements or motifs might also be important for transactivation caused by *AcMYB10*. In addition, the transcriptional activation ability of *AcF3GT*/*AcLDOX* + 35S:AcMYB10 + 35S:AcbHLH42 was much higher than that of *AcF3GT*/*AcLDOX* + 35S:AcMYB10 or *AcF3GT*/*AcLDOX* + 35S:AcbHLH42 (Figure 9B). Therefore, *AcMYB10* and *AcbHLH42* have a synergistic function in activating the promoters of *AcF3GT* and *AcLDOX*.

### 2.11. AcMYB10 Interacts with AcbHLH42 to Activate Anthocyanin Biosynthesis in N. tabacum Leafs and A. arguta Fruit

**Figure 10:**
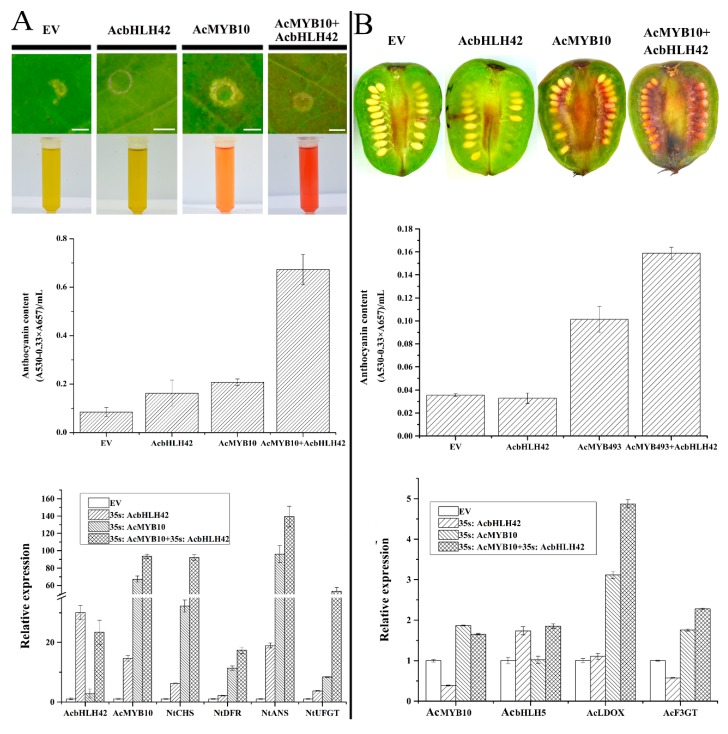
The combinatorial action of *AcMYB10* and *AcbHLH42* determines anthocyanin production in transiently expressed *N. tabacum* leaves and *A. arguta.* Color changes induced by transiently expressing either 35S:AcMYB10 or 35S:AcbHLH42 alone, or co-expressing 35S:AcMYB10 and 35S:AcbHLH42 in (**A**) *N. tabacum* leaves and (**B**) *A. arguta* fruit. The liquid in test tubes are fresh extracts from the individual infiltrated patches. The bar scales are 4 mm. Total anthocyanin content measured in (**A**) *N. tabacum* leaves and (**B**) *A. arguta* fruit. Anthocyanin content was determined 6 days after infiltration. Expression analysis of *AcMYB10*, *AcBHLH42,* and key anthocyanin biosynthesis genes in (**A**) *N. tabacum* leaves and (**B**) *A. arguta* fruit. Results represent the mean ± SD of three replicates.

To verify the function of *AcMYB10* and *AcbHLH42* in anthocyanin biosynthesis, their transient expression was examined in the tobacco leaf and *A. arguta* fruit. *A. tumefaciens* strain GV3101 harboring the recombinant plasmids of 35S:AcMYB10 and 35S:AcbHLH42 were syringe-infiltrated to the abaxial surfaces of expanding *N. tabacum* leaves. Red pigmentation was observed at the injection sites of 35S:AcMYB10 and co-injected 35S:AcMYB10 and 35S:AcbHLH42 six days after injection (Figure 10A). In comparison, the injection sites remained green in leaves injected with empty vector or 35S:AcBHLH42. Anthocyanin content was detected in 35S:AcMYB10 and co-injected 35S:AcMYB10 + 35S:AcBHLH42, but not in the empty vector and 35S:AcBHLH42. The anthocyanin content in co-expressing 35S:AcMYB10 and 35S:AcbHLH42 was significantly higher than that in 35S:AcMYB10. The five structural genes (*NtCHS*, *NtF3H*, *NtDFR*, *NtANS,* and *NtUFGT*) in the anthocyanin biosynthesis pathway showed highest expression in co-injected 35S:AcMYB10 and 35S:AcbHLH42 (Figure 10A).

To demonstrate the function of *AcMYB10* and *AcbHLH42* in the fruit tissue of kiwifruit, the same vectors were injected into the fruit of *A. arguta* cv. Baobeihong (Figure 10B). The highest levels of anthocyanins were detected in the fruits co-overexpressing *AcMYB10* and *AcbHLH42*, which corresponded to the increased expression levels of *AcMYB10*, *AcbHLH42*, *AcF3GT,* and *AcLDOX*. Thus, *AcMYB10* might accelerate anthocyanin biosynthesis through interacting with *AcbHLH42* in the fruit tissue of kiwifruit.

## 3. Materials and Methods

### 3.1. Plant Materials, Treatment, and Nucleic Acid Extraction

The kiwifruit callus was induced from *Actinidia chinensis* cv. Hongyang as previously described [42,43]. In the light and temperature treatment experiment, the callus was incubated under the following conditions: 16 °C /light (intensity of 80 μmol m^−2^ s^−1^), 24 °C/light, 32 °C/light, 16 °C/dark, 24 °C/dark, and 32 °C/dark. The callus was sampled at 0, 4, 8, 12, 16, 20, and 24 days after treatment.

In the VIGS experiment, *Agrobacterium* was injected into the fruit of ‘Hongshi No. 2′. In the transient expression experiment, *Agrobacterium* was injected into the fruit of *A. arguta* cv. Baobeihong, a purple *A. arguta* var. *purpurea* cultivar. The samples of ‘Hongshi No. 2′ and ‘Baobeihong’ were collected from a germplasm garden at Wufeng mountain, Enshi city, Hubei province.

The genomic DNA used in this study was extracted from ‘Hongyang’ leaves using a DNA extraction kit (DN38, Aidlab, Beijing). Total RNA was extracted using HiPure Plant RNA kits (R4151-03, Magen, Shanghai). The cDNA libraries were constructed by using HiScript II QRT SuperMix (R223-01, Vazyme, Nanjing).

### 3.2. Genome-Wide Analysis and Expression Profile of Kiwifruit R2R3-MYB Genes

The hidden Markov model (HMM) profile for the MYB binding domain (PF00249) was downloaded from Pfam (http://pfam.sanger.ac.uk/) and subsequently exploited to identify MYB genes from the ‘Red5′ kiwifruit genome using HMMER 3.2. The default parameters were adopted, and the cutoff value was set to 0.01. To confirm the core MYB domains, putative MYB sequences were further examined using a conserved domain search (https://www.ncbi.nlm.nih.gov/Structure/cdd/wrpsb.cgi) and InterProScan (http://www.ebi.ac.uk/interpro/). All of the raw R2R3-MYB proteins were manually inspected to generate a final MYB dataset. Subsequently, according to the identified gene IDs, the transcript sequences, coding sequences (CDS), genome sequences, chromosome locations, and annotations of these MYB superfamily proteins were extracted from the ‘Red5 genome’ [44] using Perl script. MEME Suite 5.0.5 [43] was used to identify motifs in the 155 MYB protein sequences with the following parameters: the maximum number of motifs was set to 20, and the optimum width of each motif was set from 10 to 100 residues. The chromosome location and the exon/intron structures of kiwifruit R2R3-MYB genes were plotted using the R script. Phylogenetic trees were constructed based on the conserved domain of R2R3-MYB proteins, with a bootstrap analysis of 1000 replicates using MEGA 7 software [45]. The sequences and annotation of 126 *Arabidopsis* R2R3-MYB proteins were downloaded from the TAIR (The Arabidopsis Information Resource: http://www.arabidopsis.org/). The upstream 2 kb genomic DNA sequences of anthocyanin-related AccR2R3-MYBs (labelled with a dotted line) were submitted to the Plant CARE database (http://bioinformatics.psb.ugent.be/webtools/plantcare/html/) to identify the cis-elements.

The callus, leaf, flower, and inner pericarp of ‘Hongyang’ were sampled for the RNA-seq. The transcript abundance of kiwifruit R2R3-MYB genes was calculated as fragments per kilobase of the exon model per million mapped reads (FPKM). The raw FPKM data were normalized to log2(FPKM+1) for downstream analysis. The hierarchical clustering of the expression data was constructed in the R package.

### 3.3. Quantitative Real-Time RT-PCR

RT-PCR was performed on Quant Studio 6 Flex (Life Technologies Corporation, Carlsbad, CA, USA), following the manufacturer’s instructions for AceQ qPCR SYBR Green Master Mix Q131-02 (Vazyme, Nangjing, China). The reactions were carried out as follows: 95 °C for 30 s, followed by 40 cycles at 95 °C for 10 s, 60 °C for 30 s, and 72 °C for 20 s. Melt-curve analyses were carried out as follows: 15 s at 95 °C, 1 min at 60 °C, 30 s at 95 °C, and 15 s at 60 °C. The 2^−ΔΔCt^ method was employed with *Achn107181* (Actin) as an endogenous control [46]. All of the primers used in this study are shown in Supplementary Table S4.

### 3.4. Determining the Concentration of Anthocyanin, Chlorophyll, and Flavonoid Metabolites

Total anthocyanin content was extracted and determined according to the methods described by Shin et al. [47] and Lim et al. [48]. Briefly, 0.1 g powdered tissue samples were incubated in 600 µL extraction buffer (methanol containing 1% HCl) for 6 h at 4 °C with moderate shaking. After extraction, 200 µL of water and 200 µL of chloroform were added to each sample, and absorbances were read at 530 nm and 657 nm using a microplate reader. Anthocyanin content was determined using the following equation: A530 − 0.33 × A657. Chlorophyll *a* and *b* were extracted and determined following a method described by Hiscox and Israelstam [49]. Briefly, 0.1 g powered sample was extracted with 5 mL dimethylsulfoxide for 72 h with shaking in the dark. Absorbances of 2 mL of the sample extracts were read at 663 nm and 645 nm using a spectrophotometer. Chlorophyll content was determined using the following equation: Chl *a* = (12.7 × OD 663 − 2.69 × OD645) × 5/0.1 × 1000 × 2; Chl *b* = (22.9 × OD 645 − 4.68 × OD663) × 5/0.1 × 1000 × 2. Flavonoid metabolites were extracted, isolated, and identified according to a method described by Wang et al. [50]. Briefly, 100 mg powdered sample was extracted overnight at 4 °C with 1.0 mL 70% aqueous methanol. Following centrifugation (10,000× *g* for 10 min), the extracts were passed through the SPE cartridge and filtrated before LC–MS/MS analysis. The sample extracts were analyzed using a liquid chromatography-electrospray ionization tandem mass spectrometry (LC-ESI-MS/MS) system (HPLC, UFLC SHIMADZU CBM30A system, www.shimadzu.com.cn/; MS, Applied Biosystems 4500 QTrap, www.appliedbiosystems.com.cn/). The corresponding relative metabolite contents were represented as chromatographic peak area integrals.

### 3.5. Isolation and Sequence Alignments and Transcriptional Activation Analysis of AcMYB10

Full-length DNA sequences of Acc00493 were amplificated from ‘Hongyang’ genomic DNA. PCR products were cloned into pEASY-Blunt Cloning Vector, and then the recombinant plasmid was transformed into *Escherichia coli* (trans-T1) cells using pEASY-Blunt Cloning Kit CB101 (Transgen, Beijing, China). Ten amplified PCR products and ten monoclonals were sequenced by Sanger sequencing. Full-length CDS of *AcMYB10* (Acc00493) and *AcBHLH42* (Acc19563) were amplified from the cDNA library of the inner pericarp of ‘Hongyang’. The full length and two truncated parts of *AcMYB10* (AcMYB10N: 1–111aa or AcMYB10C: 112–222aa) CDS were amplified and cloned to the pGBKT7 vector (pGBKT7-AcMYB10/AcMYB10N/AcMYB10C). The fusion vectors and empty vector (pGBKT7) were separately transformed to the yeast (*Saccharomyces cerevisiae*) strain *AH109,* harboring a MEL1 reporter. Transcriptional activation activity of the transformed yeast cells was determined after incubation at 30 °C for 3 days on SD/–Trp or SD/–Trp/–His/–Ade medium supplemented with 0.02 mg/mL X-α-gal CX11922 (Coolaber, Beijing, China).

### 3.6. Dual Luciferase Assay of Transiently Transformed Tobacco Leaves

The putative promoters of *AcF3GT* (Acc20131) (approximately ~1 kb upstream of the ATG), *AcLDOX* (Acc28876) (approximately ~1.5 kb upstream), and various deletions of the promoter fragments containing MRE cis-elements were PCR-amplified from ‘Hongyang’ genomic DNA. All of these fragments were inserted to the cloning site of pGreen0800-LUC using In-Fusion cloning CU201-02 (TransGen, Beijing, China), and were then transformed to *A. tumefaciens* GV3101. The CDSs of *AcMYB10* and *AcBHLH42* were respectively cloned to the pCAMBIA1301 over-expression vector driven by the 35S promoter using In-Fusion cloning CU201-02. Agrobacterium infiltration and measurement of luciferase activity were conducted as described previously [51,52].

### 3.7. Gene Functional Study Using the VIGS System in Kiwifruit and Over-Expression of AcMYB10 in A. chinensis, A. arguta, and N. tabacum

The 400–666 fragment of *AcMYB10* was amplified and inserted to pTRV2 to generate TRV2:AcMYB10 vector. *A. tumefaciens* strain GV3101 containing TRV1 + TRV2:AcMYB10 or TRV1 + TRV2 was injected to the fruit of ‘Hongshi No. 2′ at about 100 days after pollination (DAP) from the fruit lateral surface at four opposite points around the central section. The detailed experimental procedure is presented in [53].

Fresh calluses of ‘Hongyang’ were inoculated with *A. tumefaciens* strain *EHA105* containing the 35S:AcMYB10. A high-efficiency transformation procedure for kiwifruit was performed, as previously reported [54]. The regenerated calluses were selected on medium containing 75 mg/L of G418 and transferred to fresh selection medium for bud induction under 12:12 (light: dark) conditions at 26 °C.

The *A. tumefaciens* GV3101 strains containing 35S:AcMYB10, 35S:AcBHLH42, 35S:AcMYB10 + 35S:AcBHLH42, and the empty vector were injected into the abaxial leaf surface of six-week-old *N. tabacum* and 50 DAP fruits of *A. arguta* cv. Baobeihong, respectively. The phenotype was observed and sampled 6 days after injection.

### 3.8. Subcellular Localization Analysis

The CDSs of *AcMYB10* and *AcbHLH42* (without the stop codon) were cloned to the 101YFP (yellow fluorescent protein) vector under the control of the CaMV35S promoter. The fusion construct (AcMYB10-YFP and AcBHLH42-YFP) or the control vector (YFP), as well as cell nucleus marker VirD2NLS fused to mCherry, was transformed to *A. tumefaciens* strain GV3101 and co-infiltrated to the leaves of *N. benthamiana* with the suspension, as previously described [55]. After 48 h incubation at 25 °C, tobacco leaves were used for YFP and RFP fluorescence signal observations using a laser-scanning confocal microscope TCS-SP8 (Leica-Microsystems, Wetzlar, Germany).

### 3.9. Yeast Two-Hybrid (Y2H) Assays

The full lengths of *AcMYB10* and *AcbHLH42* CDS were respectively inserted to the pGADT7 vector, which were designated as “AD-AcMYB10” and “AD-AcMYB10” constructs in the present study. To generate binding domain (BD) constructs, the following fragments were amplified: full-length *AcMYB10*, N-terminal region (AcMYB10N:1-111aa), C-terminal region (AcMYB10C:112-222aa), and *AcBHLH42*. The amplified fragments were ligated to the pGBKT7 vector. AD and BD constructs were co-transformed into yeast strain *AH109* according to the manufacturer’s instructions (SK2400-200T, Coolaber, Beijing, China). The co-transformants were initially selected on synthetic dropout medium lacking Leu and Trp (SD/−Leu/−Trp) and were replicated on quadruple-dropout medium deficient in Ade, His, Leu, and Trp (SD/−Ade/−His/−Leu/−Trp). They were then supplemented with 15 mM 3-amino-1,2,4-triazole (3AT) CA1311 (Coolaber, Beijing, China), which is a competitive inhibitor of the HIS3 gene product. X-α-gal was also used to assess β-galactosidase activity and confirm positive interactions.

## 4. Conclusions

This study comprehensively analyzed R2R3-MYB TFs in the kiwifruit genome, demonstrating the synergistic effect of light and temperature on anthocyanin accumulation in kiwifruit. This study also showed that *AcMYB10* and *AcbHLH42* are interacting partners that contribute to anthocyanin biosynthesis by activating the transcription of *AcLDOX* and *AcF3GT*. Our study provides a basis for the functional study of R2R3-MYBs in kiwifruit, presenting new insights on the physiological mechanism and regulatory function of *AcMYB10*, as well as valuable knowledge that could be used to enhance cultivation practices and improve the quality of kiwifruit.

## Supplementary Materials

Supplementary materials can be found at https://www.mdpi.com/1422-0067/20/20/5228/s1.

## Abbreviations

HMM	Hidden Markov Model
CDS	coding sequences
FPKM	Fragments per kilobase of transcript per million mapped reads
VIGS	Virus-induced gene silencing
qRT-PCR	Quantitative real-time PCR
DAP	days after pollination
YFP	yellow fluorescent protein
RFP	red fluorescent protein
3AT	3-amino-1,2,4-triazole
CRE	Cis-acting regulatory element
LTR	Low-temperature stress
MRE	MYB-recognizing element
