# Supplementary figures and images for: Light- and Temperature-Induced Expression of an R2R3-MYB Gene Regulates Anthocyanin Biosynthesis in Red-Fleshed Kiwifruit
Source: Int J Mol Sci. 2019 Oct 22;20(20):5228. doi: 10.3390/ijms20205228 (PMC6829553; doi:10.3390/ijms20205228)

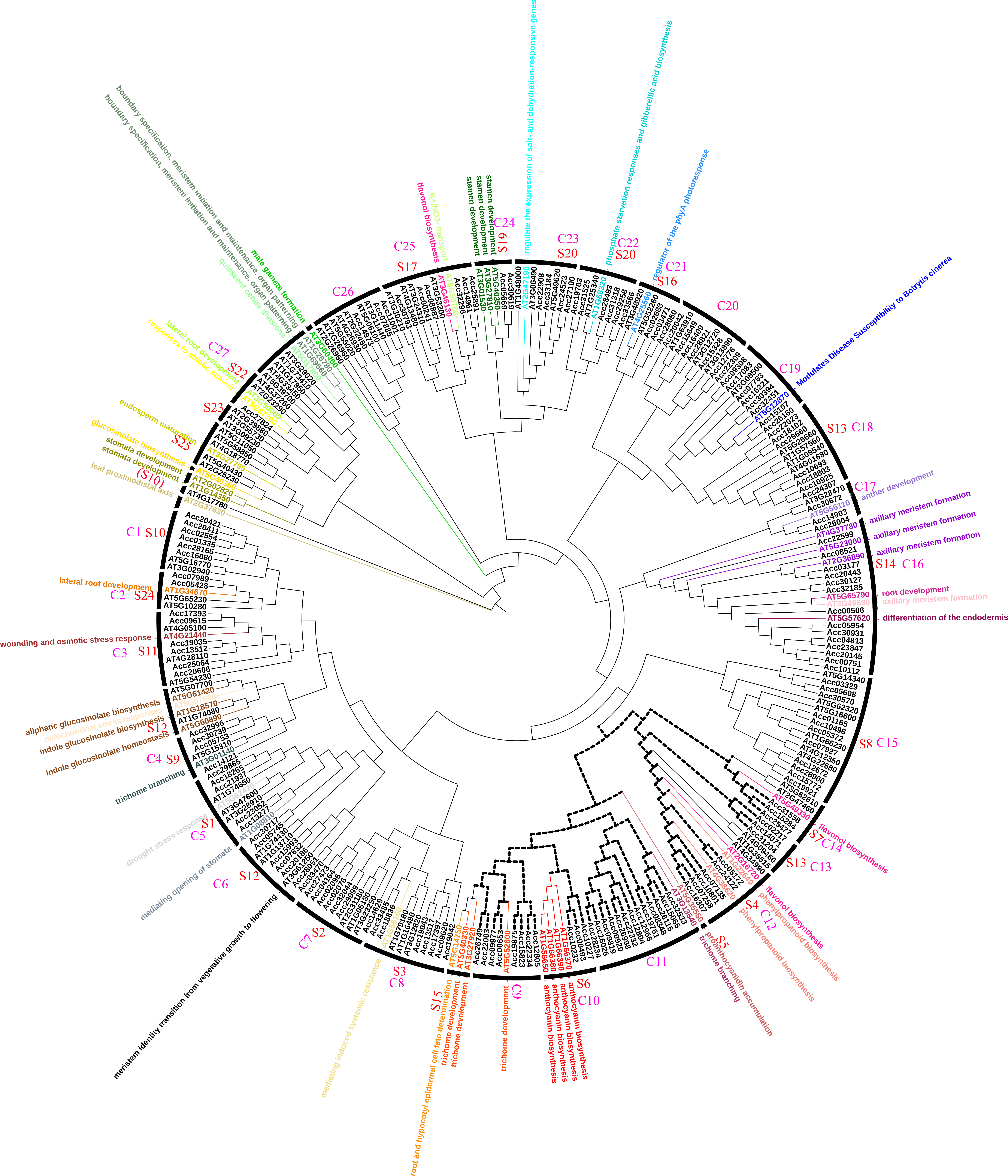

Supplement: Supplementary file 1 [file ijms-20-05228-s001.zip › Supplementary material/Supplementary Figure1-8TIFF/Supplementary Figure 3.tif]

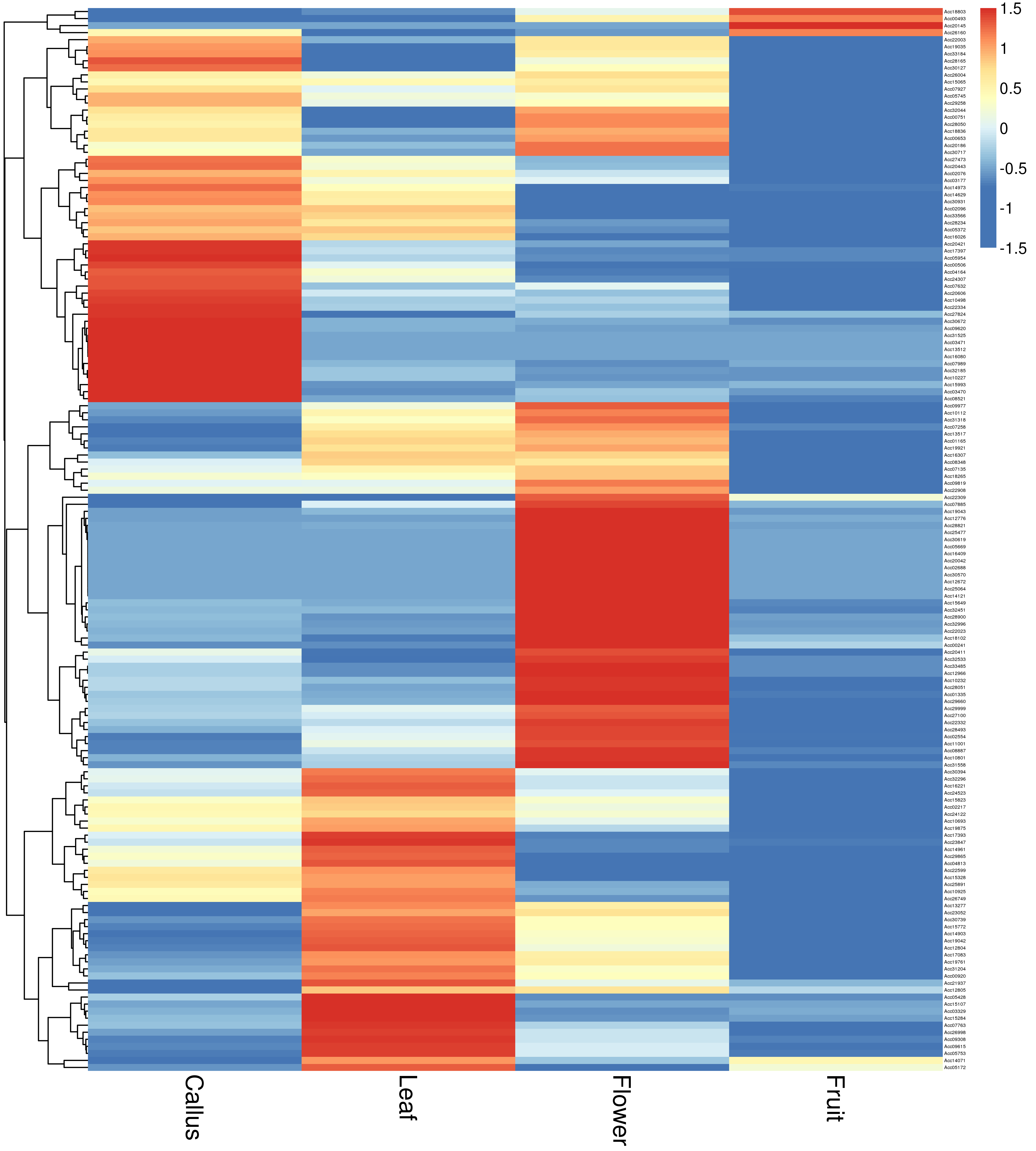

Supplement: Supplementary file 1 [file ijms-20-05228-s001.zip › Supplementary material/Supplementary Figure1-8TIFF/Supplementary Figure 4 .png]

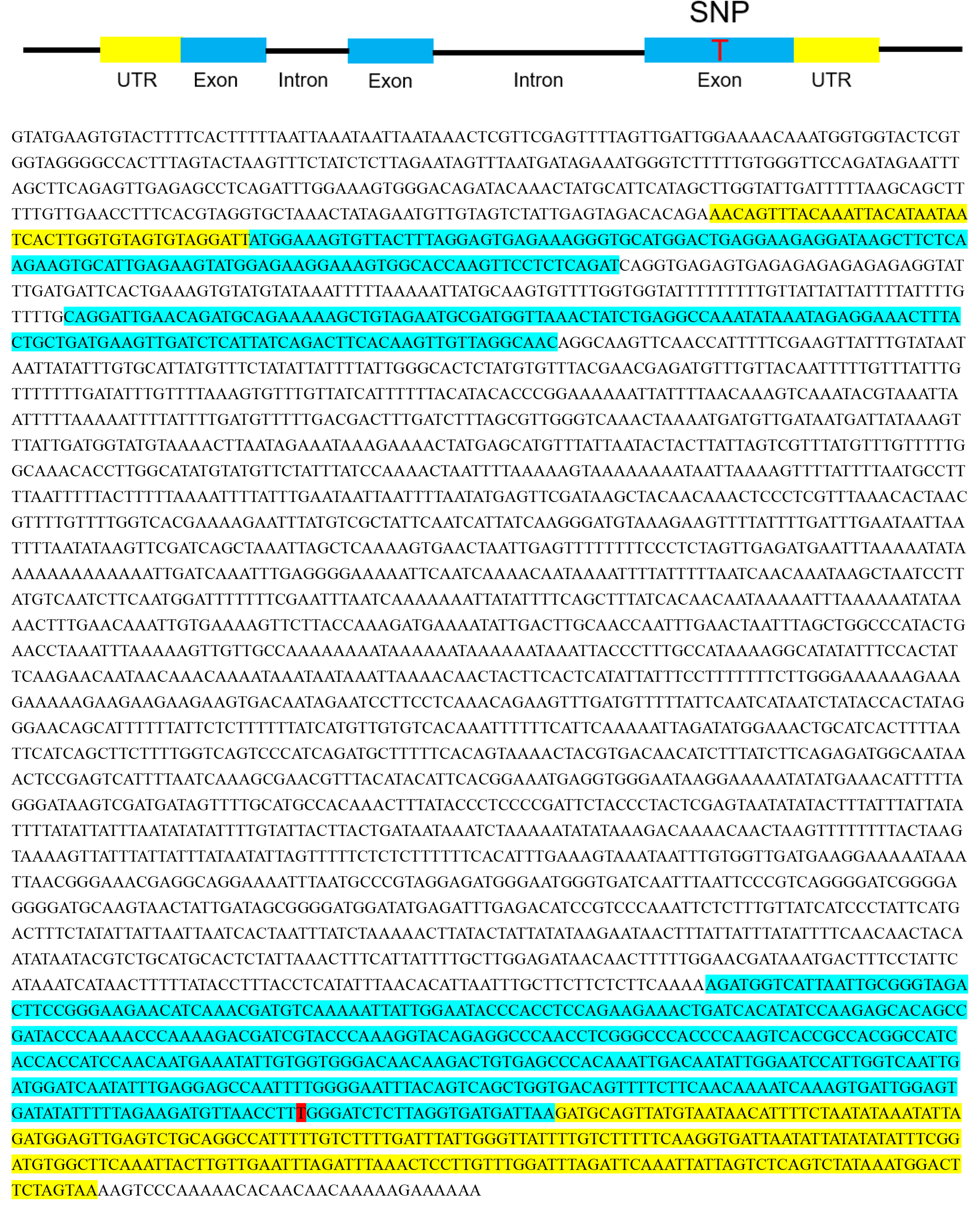

Supplement: Supplementary file 1 [file ijms-20-05228-s001.zip › Supplementary material/Supplementary Figure1-8TIFF/Supplementary Figure 5.tif]

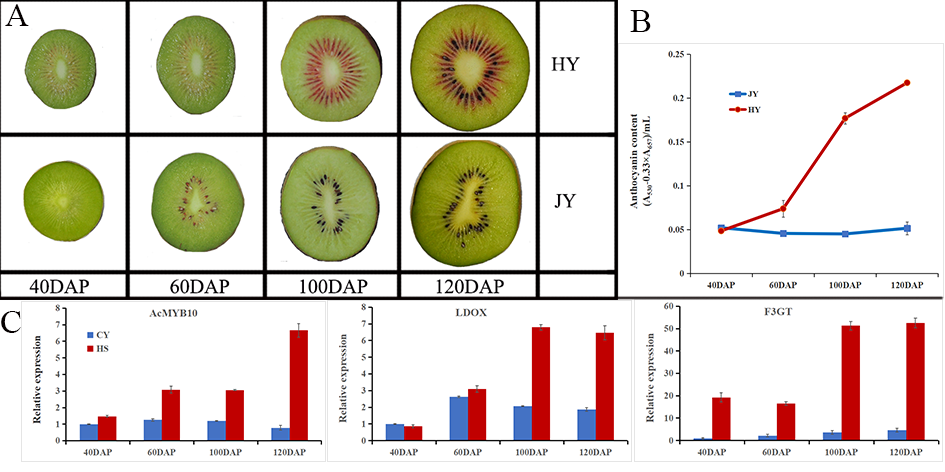

Supplement: Supplementary file 1 [file ijms-20-05228-s001.zip › Supplementary material/Supplementary Figure1-8TIFF/Supplementary Figure 6.tif]

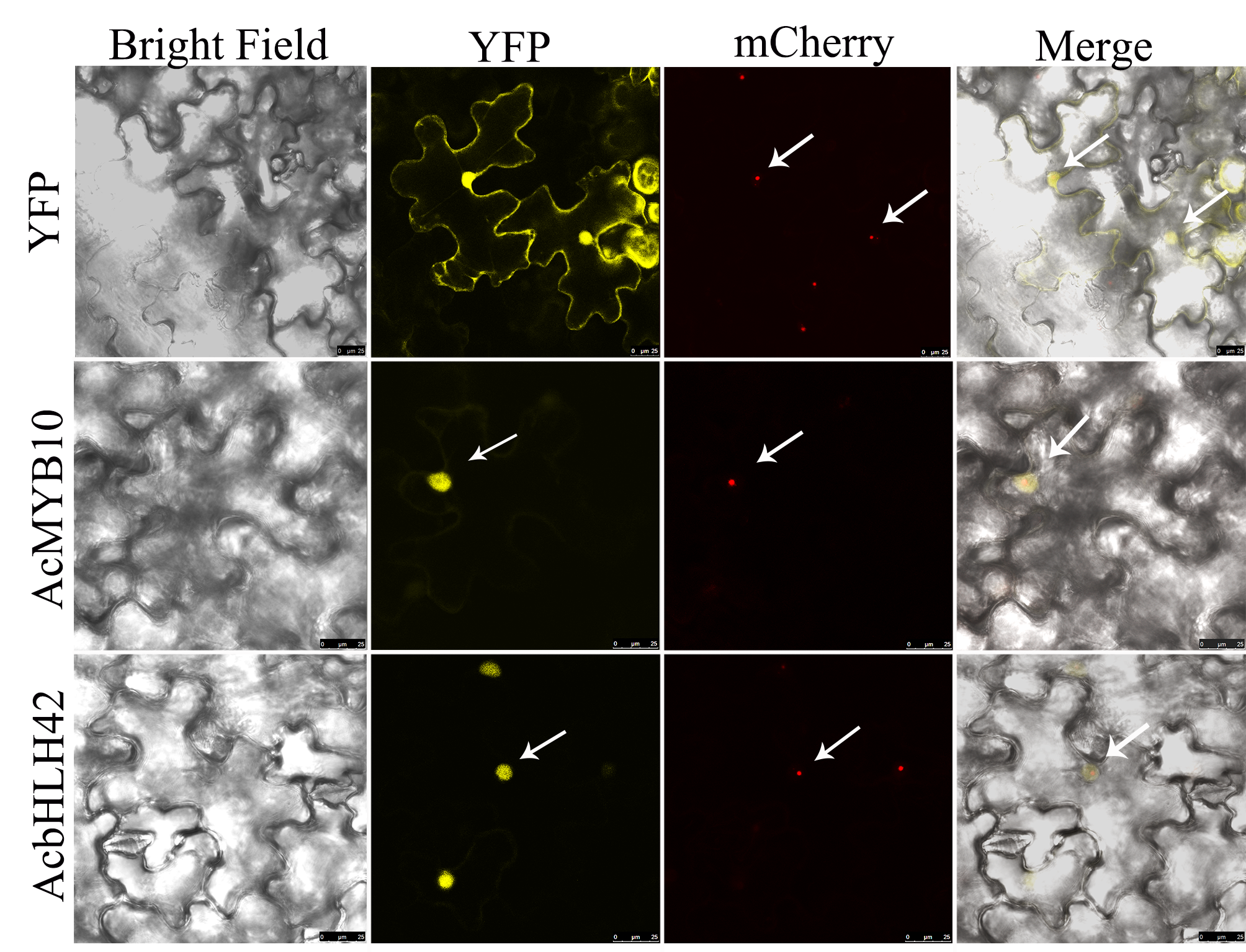

Supplement: Supplementary file 1 [file ijms-20-05228-s001.zip › Supplementary material/Supplementary Figure1-8TIFF/Supplementary Figure 7.tif]

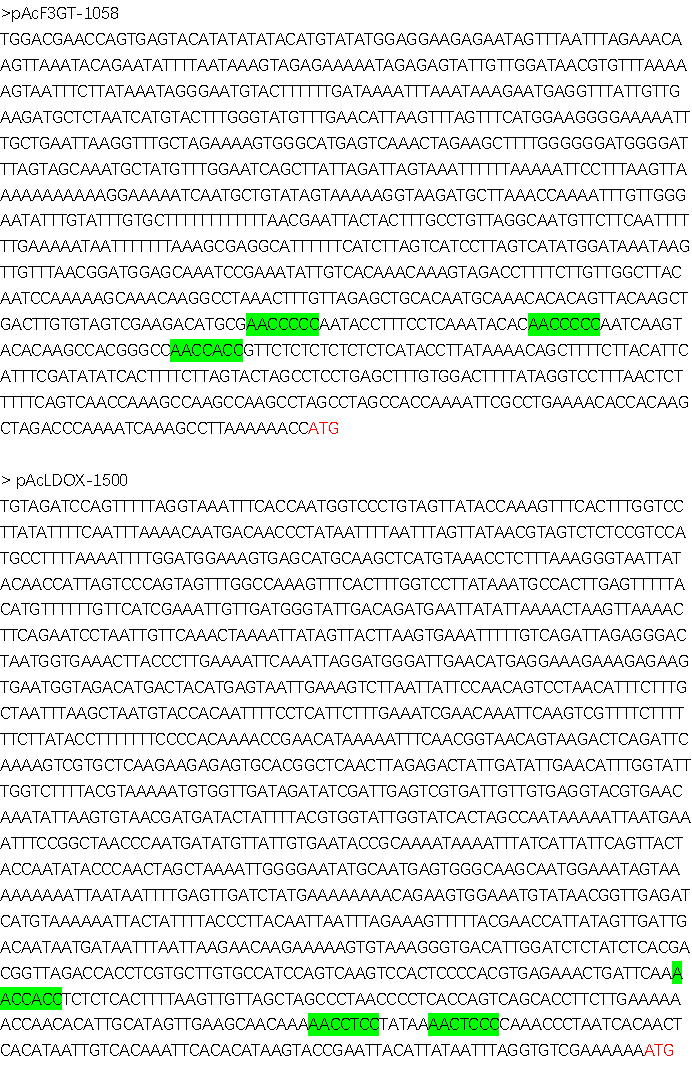

Supplement: Supplementary file 1 [file ijms-20-05228-s001.zip › Supplementary material/Supplementary Figure1-8TIFF/Supplementary Figure 8.tif]
