# Supplementary material for: Light- and Temperature-Induced Expression of an R2R3-MYB Gene Regulates Anthocyanin Biosynthesis in Red-Fleshed Kiwifruit
Source: Int J Mol Sci. 2019 Oct 22;20(20):5228. doi: 10.3390/ijms20205228 (PMC6829553; doi:10.3390/ijms20205228)
Supplement: Supplementary file 1 [file ijms-20-05228-s001.zip › Supplementary material/Supplementary Figure1-8-legend.doc]

**Supplementary Figure 1.** Chromosomal distribution and sequence logos within AccR2R3-MYB domains.

(A) Sequence logos of the R2 and R3 MYB repeats for the full-length alignments of all AccR2R3-MYB domains. The bit score indicates the information content for each position in the sequence. The positions of the three α-helices are marked (Helix 1 to 3). The asterisks indicate the conserved tryptophan residues (W) in the MYB domain. (B) Chromosomal locations of AccR2R3-MYBs. The chromosomal position of each AccR2R3-MYB was mapped according to the ‘Red5’ genome. The chromosome number is indicated at the bottom of each chromosome. The scale is in megabases (Mb).

**Supplementary Figure 2.** Phylogenetic relationships, architecture of conserved protein motifs, and gene structure in AccR2R3-MYB TFs.

(A) The neighbor-joining (NJ) tree on the left includes the 155 AccR2R3-MYB full protein sequence. (B) Distribution of conserved motifs within the AccR2R3-MYB amino acid sequences as determined by the MEME tool. (C) Exon/intron structures of AccR2R3-MYBs. Exon(s) and intron(s) are represented by yellow boxes and black lines, respectively.

 **Supplementary** **Figure 3.** Phylogenetic tree of the AccR2R3-MYB proteins and AtR2R3-MYB proteins

The circular unrooted tree was generated by the neighbor-joining method using the *Arabidopsis* R2R3-MYB genes as a reference. The functional members and clades are highlighted with different colors or bold dotted lines.

**Supplementary** **Figure 4.** Tissue expression profiles of 155 AccR2R3-MYB genes in kiwifruit.

(A) Hierarchical clustering of expression profiles of 155 AccR2R3-MYBs in different tissues. Log2(FPKM+1) values were displayed according to the color code (top right).

**Supplementary Figure 5.** The full-length DNA sequence of Acc00493 amplificated from ‘Hongyang’ genomic DNA.

Yellow highlight marks UTR, blue highlight marks exon, red highlight marks the SNP between *AcMYB75* and *AcMYB10*.

**Supplementary** **Figure 6.** Expression of *AcMYB10*, *AcF3GT,* and *AcLDOX* during the development of *A. chinensis* cv. Hongyang and *A. chinensis* cv. Jinyan

(A) Bisected fruit across four developmental stages. (B) The content of anthocyanin in ‘Hongyang’ and ‘Jinyan’ during fruit development. (C) Verification of the expression levels for candidate anthocyanin genes. Results represent the mean ± SD of three replicates.

**Supplementary** **Figure 7.** Subcellular localization of *AcMYB10* protein and *AcBHLH42* protein.

Confocal images showing yellow (for YFP) and red (for mCherry) fluorescence signals in the epidermal cells of the infiltrated tobacco (*N. benthamiana*) leaves. Tobacco leaves were agroinfiltrated with YFP (yellow fluorescent protein) empty control or AcMYB10-YFP fusion protein and AcBHLH42-YFP fusion protein, along with a nuclear marker gene VirD2NLS fused to mCherry.

**Supplementary** **Figure 8.** The MREs (green color) in the promoters of *AcLDOX* and *AcF3GT*. The green highlight shows the putative MYB recognizing element.
